# Effect of single-dose diode laser photobiomodulation on orthodontic pain following initial archwire placement: a randomized clinical trial

**DOI:** 10.1186/s12903-025-06326-2

**Published:** 2025-07-01

**Authors:** Amin Golshah, Azadeh Kazemisaleh, Fatemeh Azizi, Amin Hossein Nejad

**Affiliations:** 1 Department of Orthodontic, School of Dentistry, Kermanshah University of Medical Sciences, Shariati Street, Kermanshah, 67139546581 Iran; 2 General Dentistry, School of Dentistry, Kermanshah University of Medical Sciences, Kermanshah, Iran

**Keywords:** Photobiomodulation, Lasers, Pain, Orthodontic wires

## Abstract

**Background:**

This study assessed the effect of photobiomodulation (PBM) through single-dose diode 940 nm laser on pain after initial archwire placement.

**Methods:**

This parallel-design clinical trial with 1:1 allocation ratio was conducted on 120 orthodontic patients with Class I molar relationship and 3–6 mm crowding, or overbite. Orthodontic brackets had been previously placed by an orthodontist. The patients were randomly assigned to three groups (*n* = 40) of PBM, placebo (laser in off mode), and control (no intervention). Right after initial archwire placement, patients in PBM group underwent 940 nm laser irradiation with 32 J/cm^2^ energy density in contact mode, and five areas were irradiated. Laser in off mode was used for the placebo group. The level of pain experienced by patients after archwire placement was recorded subjectively by using a visual analog scale (VAS). The Pain Catastrophizing Scale (PCS) was filled out by patients after 7 days to assess the correlation of personal traits with actual pain. Data were analyzed by the Chi-square test, independent t-test, ANOVA, and ANCOVA (alpha = 0.05).

**Results:**

Type of intervention had no significant effect on VAS pain score (*P* = 0.654) although slightly lower pain was recorded in the PBM group. Location of orthodontic wire placement (maxilla/mandible) had no significant effect on the pain score (*P* = 0.780), although the mean pain score in the mandible was slightly higher than that in the maxilla.

**Conclusion:**

In the present study, PBM delivered using a single-session 940 nm diode laser had no significant effect on pain after initial placement of orthodontic archwires.

**Trial registration:**

Trial registration number: IRCT20230603058377N1. Retrospectively registered. Registration date: 08/07/2023.

## Introduction

Orthodontic science deals with the treatment of dental and maxillofacial anomalies [1]. Orthodontic treatment improves leveling and alignment of teeth and masticatory function through application of mechanical forces [2]. Orthodontic force application results in development of edema and ischemia in the periodontal tissue, which trigger inflammatory reactions and subsequent production of pain mediators such as cytokines and prostaglandins. As a result, orthodontic treatment is often associated with pain [2]. Evidence shows that the initial orthodontic pain is associated with elevated levels of prostaglandin E2 [3]. Pain perception is a cognitive process that is initiated by stimulation of nerve terminals [4]. A previous study reported that of patients who underwent fixed orthodontic treatment, 91% experienced pain and 39% had pain during all phases of treatment [5]. Orthodontic pain is an unwanted complication which can cause some concerns for patients and can even lead to treatment discontinuation [3]. Also, pain can impede oral hygiene practice [6]. Pain associated with initial archwire placement can significantly impair mastication and biting of foods, and cause gingival inflammation and tooth hypersensitivity in 50% of patients [2].

Efficient pain management plays a pivotal role in increasing patient satisfaction and cooperation during orthodontic treatment [7]. Orthodontic patients may experience different levels of pain following placement of initial archwires [7]. Upon placement of initial archwires, pain increases in the first 24 h at the site of archwire placement, and then subsides within one week [3, 6–8]. Several methods have been proposed for pain reduction [7] such as analgesic intake [9], vibrational forces for the PDL, and transcutaneous electrical nerve stimulation [5, 10, 11]. Such methods have shown variable levels of success, and have some shortcomings [9]. Non-steroidal anti-inflammatory drugs (NSAIDs) such as ibuprofen are currently administered for pain relief in orthodontic patients [9]. NSAIDs inhibit the prostaglandin synthesis pathway by inhibition of the activity of cyclooxygenase enzyme, and alleviate pain as such [9]. However, some concerns exist regarding the deceleration of orthodontic tooth movement by administration of NSAIDs [7]. Also, intake of NSAIDs may be associated with side effects such as gastrointestinal problems, coagulopathies, congestive heart disease, and allergic reactions [9].

Photobiomodulation (PBM) through 940 nm diode laser is a non-pharmaceutical modality for orthodontic pain reduction [12, 13]. Dental lasers currently have several applications and are used for caries detection [14], oral surgical procedures [15], cavity preparation [16], and enamel conditioning [17]. Dental lasers are also used in orthodontics for the purpose of PBM [18]. PBM has insignificant side effects, and has been suggested for pain reduction in orthodontics [19]. Over 700 randomized clinical trials have been published so far regarding the therapeutic efficacy of PBM therapy for different conditions [20, 21]. PBM therapy is a novel therapeutic modality based on irradiation and absorption of light in red or near infrared wavelengths [22]. The optimal efficacy of PBM for reduction of orthodontic pain, enhancement of orthodontic tooth movement, and acceleration of tissue healing has been previously reported [7, 23, 24].

Some studies indicated that PBM decreased pain due to initial orthodontic archwire placement in the first days after installation [6, 25]; however, some others refuted this effect [26, 27]. Considering the existing controversy on this topic, this study aimed to assess the effect of PBM through single-dose laser-based PBM on pain during initial orthodontic archwire placement. The null hypothesis of the study was that PBM through single-dose 940 nm diode laser would have no significant effect on pain during initial orthodontic archwire placement.

## Methods

This study was conducted at the Orthodontics Department of School of Dentistry, Kermanshah University of Medical Sciences between October 2022 and December 2022. The study protocol was approved by the ethics committee of the university (IR.KUMS.REC.1402.074), and registered in the Iranian Registry of Clinical Trials (IRCT20230603058377N1).

### Trial design

A parallel-design randomized controlled clinical trial with 1:1 allocation ratio was designed in which the experimental group received PBM, the placebo group received laser in off mode, and the control group received no intervention. The study adheres to Consolidated Standards of Reporting Trials (CONSORT) guidelines.

### Participants, eligibility criteria, and settings

The inclusion criteria were orthodontic patients with Class I molar relationship, 3–6 mm crowding [28], or overbite who had orthodontic brackets for 0.014-inch nickel-titanium archwire placed by an orthodontist for fixed orthodontic treatment [29], were between 13 and 21 years, had all the permanent teeth except for third molars, required maxillary and mandibular orthodontic treatment with the same bracket type, and were willing to participate in the study.

The exclusion criteria were patients with low pain perception threshold showing severe or unseal pain upon testing by an algometer, systemic intake of medications, temporomandibular disorders, impacted teeth or periodontal problems, intake of analgesics during the study period, use of head gear and mini-screws, patients with systemic underlying diseases, smoking, alcohol consumption, history of antibiotic therapy in the past 6 months, history of periodontal therapy in the past year, plaque index > 4, crowding > 6 mm, and unwillingness for study enrollment. Patients with severe pain during the study were also excluded (those expressing mild pain remained in the study).

The sample consisted of 120 orthodontic patients presenting to an orthodontic clinic in Kermanshah, Iran, who were selected by convenience sampling.

### Interventions

After obtaining written informed consent from the patients or their legal guardians, the pain perception threshold of patients was measured by an algometer (Baseline, Fabrication Enterprises, Inc., New York, NY). For this purpose, the device applied pressure to a body part until the patient felt pain. Those with very low pain perception threshold or unusual or severe reaction were excluded. The patients were then randomly assigned to three groups (*n* = 40) of PBM, placebo (laser in off mode), and control (no intervention).

In the laser group, a diode laser (Epi X, Biolase, USA) with a 940 nm wavelength was used. Initially, five patients underwent pilot laser irradiation sessions lasting up to 220 s to determine the minimum effective dose for significant pain reduction. This dose was then selected for the main trial. PBM was applied immediately after archwire placement. Both patients and clinicians wore standard protective eyewear during the procedure. The teeth were dried using an air spray, and the laser was applied to the buccal and lingual root surfaces for 180 s total—90 s per side—at an energy density of 32 J/cm² and a power of 300 mW in continuous mode. Irradiation was performed in contact mode using the deep tissue handpiece without a spacer, with the spot size in full contact with the mucosa approximately 1.7 cm². The laser beam was directed from the cervical region toward the apex of each tooth. In the placebo group, the same process was repeated but with laser in off mode. In the control group, no intervention was performed after archwire placement. Archwire placement was performed by an orthodontist for all patients. Al patients were then asked to fill out the visual analog scale (VAS) questionnaire right after initial archwire placement in the clinic. The VAS used for subjective pain assessment was a 10-cm line printed on a piece of paper with 0 indicating no pain and 10 indicating worst pain imaginable. The patients were asked to express their pain level by selecting a number from 0 to 10. Scores 0–3 indicated mild pain, scores 4–7 indicated moderate pain, and scores 8–10 indicated severe pain [13, 30].

To assess the correlation of personal traits with actual pain, the Pain Catastrophizing Scale (PCS) was also filled out by patients at home 7 days after initial archwire placement. It has three domains of rumination, helplessness, and magnification. Each item is scored using a 5-point Likert scale with 4 indicating always and 0 indicating never. It is a self-reported questionnaire with 13 items. Lower total scores indicate lower catastrophizing of pain and vice versa [31].

The patients were refrained from taking any medication, and were instructed to only consume 500 mg acetaminophen in case of severe pain and inform the researcher about it.

### Outcomes (primary and secondary)

The main objective of this study was to assess the effect of PBM on pain after initial orthodontic archwire placement.

### Sample size calculation

The sample size was calculated to be 120 patients (*n* = 40 in each group) according to a previous study by Qamruddin et al. [4], assuming the standard deviation of pain score to be 3.1 in the placebo and 2.9 in the PBM group, accuracy (d) of 2.5, percentage of dropouts (f) to be 0.2, alpha = 0.05, and study power of 90%.

### Interim analyses and stopping guidelines

No interim analyses were performed, and no stopping guidelines were established.

### Randomization

The patients were randomly assigned to three groups by permuted block randomization with classification based on gender. The randomization sequence was determined by a statistician using the RANDOM feature of Excel software and the Sort command separately for each level and A, B and C codes. To prevent prediction of the group of the last patient in each block, blocks with different sizes of 3 and 6 in all levels were used. Randomization at each level was performed for 33 patients.

### Allocation concealment

Sequentially numbered, opaque sealed envelopes were used for this purpose. The coding of the groups was written on pieces of paper, which were wrapped in aluminum foils and placed in envelopes according to the previously generated random sequence. Totally, 40 envelopes were considered for the female, and 26 for the male category. Number and category were written on all envelopes (e.g., 1 M indicated first patient in the male category). A copying paper was also placed in the envelopes that copied the number and category written on the back of the envelopes. Two plastic containers were considered for the two categories of male and female, and the respective envelopes were placed in them according to their number. The envelopes were prepared by a dental student blinded to the group allocations. Upon inclusion of patients, the assistant not informed about the group allocations randomly selected one envelope according to the gender of patients and delivered the envelope to the orthodontist. Accordingly, the orthodontist selected the pain control method for the respective patient.

### Blinding

To ensure blinding, the audible signal of the laser device was disabled, so the patient and the operator could not distinguish between the active and placebo modes, especially since the 940 nm wavelength is not visible.

### Statistical analysis

Normal distribution of data was confirmed by the Kolmogorov-Smirnov test. Thus, the Chi-square test, independent t-test, ANOVA, ANCOVA and post hoc gender analysis were applied for the comparisons using SPSS version 20 (SPSS Inc., IL, USA) at 0.05 level of significance.

## Results

### Participant flow

**Fig. 1:**
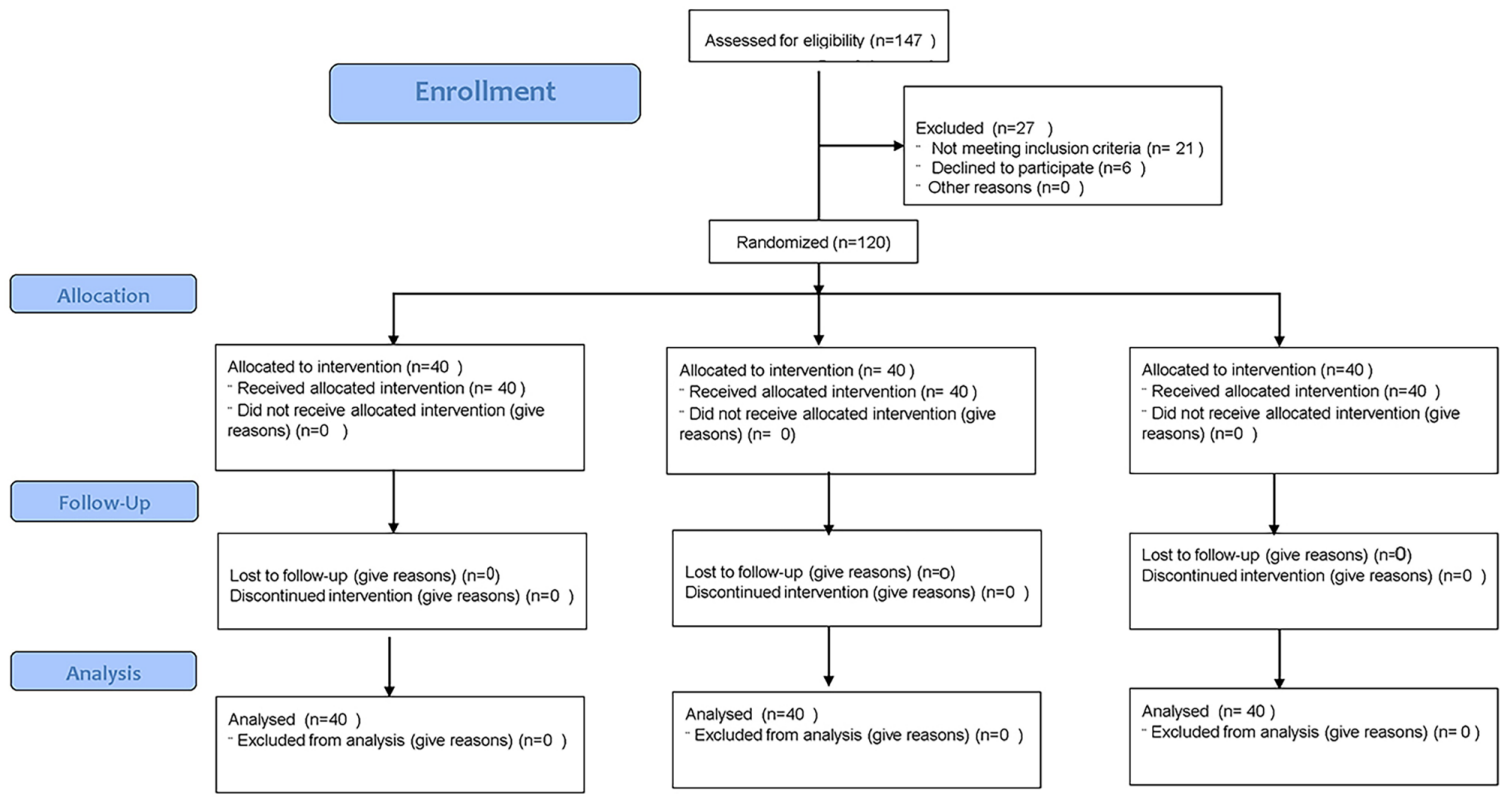
CONSORT flow-diagram of patient selection and allocation

Initially, 147 patients were recruited; of which, 27 were excluded since 21 met the exclusion criteria and 6 were not willing to participate in the study. Figure 1 shows the CONSORT flow-diagram of patient selection and allocation. The final sample consisted of 120 patients including 31 males (25.8%) and 89 females (74.2%), with a mean age of 17.22 ± 4.53 years. None of the patients used acetaminophen for pain relief. Table 1 presents the age and gender distribution of patients in the three groups. As shown, the three groups had no significant difference in gender (*P* = 0.568) or age distribution (*P* = 0.403).

**Table 1:** Age and gender distribution of patients in the three groups

		Method			*P*-value
		Control	Laser off	Laser on	
Sex	Male	12(30.0%)	8(20.0%)	11(27.5%)	0.568^†^
	Female	28(70.0%)	32(80.0%)	29(72.5%)	
Age		16.42 ± 4.34	17.60 ± 4.94	17.62 ± 4.28	0.403^‡^

Mean ± standard deviation and count (percentage %) are presented for continuous and categorical data, respectively

^†^ One-way ANOVA

^‡^ Chi-square test

### Harms

No patients were harmed during the study.

### Subgroup analyses

Primary outcome: Table 2 presents the mean VAS pain score in the three groups. ANCOVA was applied to assess the effect of intervention (group) and location of archwire placement (maxilla/mandible) adjusted for rumination, helplessness, and magnification according to the PCS results on the VAS pain score (Table 3). The results showed that type of intervention had no significant effect on the VAS pain score (*P* = 0.654) although the mean pain score in the PBM group was slightly lower than that in the other two groups. Location of archwire placement had no significant effect on VAS pain score either (*P* = 0.780) although the mean VAS pain score in the mandible was slightly higher than that in the maxilla.

**Table 2:** Mean VAS pain score in the three groups and ANCOVA test for the effect of intervention (group) and location of archwire placement (maxilla/mandible) adjusted for rumination, helplessness, and magnification on pain score

		Group									*P*-value
		Control			Placebo			PBM			
		Count	Mean	SD	Count		Mean	SD	Count	Mean	
Jaw	Maxilla	20	3.35	2.30	20	3.60	2.41	20	3.10	2.00	0.780
	Mandible	20	3.90	2.25	20	3.95	2.31	20	3.70	3.10	
	Total	40	3.62	2.26	40	3.78	2.34	40	3.40	2.59	
*P*-value		0.654									

Abbreviation: SD, Standard deviation

**Table 3:** Results of ANCOVA for the effect of intervention (group) and location of archwire placement (maxilla/mandible) adjusted for rumination, helplessness, and magnification on pain score

Source	Type III Sum of Squares	df	Mean Square	F	*P*-value	Partial Eta Squared	Observed Power
Corrected Model	190.544	6	31.757	7.380	0.000	0.282	1.000
Intercept	280.831	1	280.831	65.262	0.000	0.366	1.000
Method	3.665	2	1.832	0.426	0.654	0.007	0.118
Jaw	0.337	1	0.337	0.078	0.780	0.001	0.059
Rumination	10.348	1	10.348	2.405	0.124	0.021	0.337
Helplessness	21.197	1	21.197	4.926	0.028	0.042	0.595
Magnification	1.893	1	1.893	0.440	0.508	0.004	0.101
Error	486.256	113	4.303				
Total	2232.000	120					
Corrected Total	676.800	119					

A post hoc gender analysis revealed no significant difference in VAS scores between males and females (*P* = 0.461), consistent with earlier literature suggesting that gender may not significantly influence orthodontic pain perception.

## Discussion

This study assessed the effect of photobiomodulation (PBM) through single-dose 940 nm diode laser on pain after initial orthodontic archwire placement. The null hypothesis of the study was that PBM with single-dose laser would have no significant effect on pain during initial orthodontic wire placement. The findings did not reveal statistically significant differences in pain scores between the intervention and control groups, supporting the null hypothesis. Although slightly lower pain scores were recorded in the PBM group, the location of orthodontic wire placement (maxilla/mandible) also showed no significant effect on the pain score, although pain in the mandible was marginally higher. Thus, the null hypothesis of the study was accepted.

This result aligns with findings from studies by Abtahi et al. [32] and da Cunha et al. [33], which reported non-significant effects of low-level laser therapy (LLLT), emphasizing the variability in PBM outcomes due to differences in dosimetry and study design. These findings are consistent with Prasad et al. [34], who noted that although LLLT may not show immediate analgesic effects, reduced pain was observed in later stages of treatment. In a systematic review, Eslamipour et al. [35] evaluated the effects of LLLT on orthodontic pain and showed that it decreased pain only within a period of 2 h to 3 days and no significant difference was found between the laser and control groups in the next time points. The results of the abovementioned studies were almost in line with the present findings, showing no or insignificant effect of PBM on orthodontic pain. Lack of a significant difference in pain score among the groups in the present study may be due to the fact that initial placement of archwires often causes mild pain.

Wu et al. [36] evaluated LLLT’s effects on pain and somatosensory function, indicating reduced hypersensitivity, possibly due to its effects on trigeminal nerve activity. Giudice et al. [37] and Turhani et al. [38] reported significant short-term pain relief, while Celebi et al. [28] observed reduced pain in both active and placebo groups. Fekrazad et al. [39] showed that even placebo laser application provided lower pain scores compared to no intervention. These contrasting findings suggest a placebo response or influence from the non-specific effects of intervention.

Photobiomodulation (PBM) is believed to reduce orthodontic pain by (I) decreasing inflammatory mediators such as prostaglandin-endoperoxide synthase 2 and interleukins, and (II) promoting beta-endorphin release. However, the optimal laser dosage remains undefined, and differences in energy density, wavelength, and irradiation technique further complicate comparisons across studies [4, 40–48]. While the present study used a single PBM application immediately after archwire placement, evidence suggests that peak orthodontic pain typically occurs around 24 h later. Therefore, applying PBM again at that time may yield better analgesic outcomes. Future studies should explore using multiple PBM sessions—particularly immediately post-treatment and again at 24 h—to align more closely with pain trajectory. Moreover, the analgesic response may vary between high- and low-density laser irradiation, as higher energy densities might provide more immediate relief, though excessive dosing may trigger inhibitory biological effects. Optimizing both dosage and timing is thus critical for maximizing PBM efficacy in orthodontic pain management.

To address concerns regarding differences in wavelengths and laser parameters, several recent studies provide insight. Camacho et al. [49] systematically reviewed effective wavelength ranges for orthodontic PBM, identifying diode lasers in the 810–980 nm spectrum as optimal for pain relief in active treatment phases. Similarly, Caccianiga et al. [50] emphasized that inconsistencies in reported outcomes often stem from variability in parameters such as wavelength, energy density, and application technique, calling for standardized protocols in clinical trials. Rumão et al. [51] confirmed that low-intensity diode lasers significantly reduced initial orthodontic pain, further supporting the importance of specific laser configurations. These findings strengthen the rationale for carefully selecting and reporting laser parameters in PBM trials.

In support of these issues, recent evidence has broadened the context for interpreting PBM effectiveness. Hmida et al. [52] highlighted significant pain relief with PBM at 980 nm and 600 J energy, emphasizing the critical role of laser parameters. Wang et al. [53] detailed PBM’s mechanisms at the cellular level, including ATP upregulation and anti-inflammatory pathways, proposing that repeated or targeted dosing may optimize results. These findings further reinforce the clinical importance of using well-defined laser-specific parameters rather than generalizing PBM effects across light sources.

### Limitations

This study evaluated the effects of a single-dose PBM by 940 diode laser with a short follow-up period, limiting insights into long-term outcomes. The single-institution setting and relatively homogenous patient sample may restrict generalizability.

### Generalizability

Pain perception varies individually and culturally, and findings may not fully apply to other age groups or clinical environments using differing orthodontic or laser protocols. Future studies should evaluate the effect of repeated PBM doses, longer follow-up periods, and include diverse patient groups to better assess the efficacy of PBM in orthodontic pain management.

Given the non-significant pain reduction observed, clinicians may need to weigh the costs and logistical demands of single-dose PBM against its uncertain benefits in routine orthodontic practice.

## Conclusion

In the present study, PBM delivered through a single diode laser application had no significant effect on pain during initial placement of orthodontic archwires. Despite this, the study contributes meaningful data to guide future investigations aiming to optimize PBM protocols for clinical pain management.

## Data Availability

The data used to support the findings of this study were supplied by corresponding author under license and data will be available on request. Requests for access to these data should be made to corresponding author.

## Abbreviations

LLLT: Low-Level Laser Therapy
NSAIDs: Non-steroidal Anti-Inflammatory Drugs
PBM: Photobiomodulation
PCS: Pain Catastrophizing Scale
VAS: Visual Analog Scale

## References

[1] Glineur R, Balon-Perin A. Orthodontic treatment in children and adults. Rev Med Brux. 2001;22:A299–303. 11680192

[2] He WL, Li CJ, Liu ZP, Sun JF, Hu ZA, Yin X, et al. Efficacy of low-level laser therapy in the management of orthodontic pain: a systematic review and meta-analysis. Lasers Med Sci. 2013;28:1581–9. 23001570 10.1007/s10103-012-1196-y

[3] Deana NF, Zaror C, Sandoval P, Alves N. Effectiveness of low-level laser therapy in reducing orthodontic pain: a systematic review and meta-analysis. Pain Res Manag. 2017;2017:8560652. 29089818 10.1155/2017/8560652 PMC5635293

[4] Qamruddin I, Alam MK, Abdullah H, Kamran MA, Jawaid N, Mahroof V. Effects of single-dose, low-level laser therapy on pain associated with the initial stage of fixed orthodontic treatment: a randomized clinical trial. Korean J Orthod. 2018;48:90–7. 29564218 10.4041/kjod.2018.48.2.90 PMC5854886

[5] Shenoy N, Shetty S, Ahmed J, Shenoy KA. The pain management in orthodontics. J Clin Diagn Res. 2013;7:1258–60. 23905155 10.7860/JCDR/2013/4860.3036 PMC3708250

[6] Tortamano A, Lenzi DC, Haddad AC, Bottino MC, Dominguez GC, Vigorito JW. Low-level laser therapy for pain caused by placement of the first orthodontic archwire: a randomized clinical trial. Am J Orthod Dentofac Orthop. 2009;136:662–7. 10.1016/j.ajodo.2008.06.028 19892282

[7] Farzan A, Khaleghi K. The effectiveness of low-level laser therapy in pain induced by orthodontic separator placement: a systematic review. J Lasers Med Sci. 2021;12:e29. 34733752 10.34172/jlms.2021.29 PMC8558704

[8] Marya A, Venugopal A. The use of technology in the management of orthodontic treatment-related pain. Pain Res Manag. 2021;2021:5512031. 33763158 10.1155/2021/5512031 PMC7964123

[9] Kaya Y, Alkan O, Komuroglu AU, Keskin S. Effects of ibuprofen and low-level laser therapy on orthodontic pain by means of the analysis of interleukin 1-beta and substance P levels in the gingival crevicular fluid. J Orofac Orthop. 2021;82:143–52. 33097977 10.1007/s00056-020-00254-2

[10] Bakdach WMM, Hadad R. Effectiveness of supplemental vibrational force in reducing pain associated with orthodontic treatment: a systematic review. Quintessence Int. 2020;51:742–52. 32368767 10.3290/j.qi.a44497

[11] Al Shayea EI. Comparative assessment between ibuprofen, chewing gum, and bite wafers in pain control following first archwire placement in orthodontic patients. J Contemp Dent Pract. 2020;21:416–20. 32584279

[12] Dompe C, Moncrieff L, Matys J, Grzech-Lesniak K, Kocherova I, Bryja A et al. Photobiomodulation-underlying mechanism and clinical applications. J Clin Med. 2020;9. 10.3390/jcm9061724 PMC7356229 32503238

[13] Lim HM, Lew KK, Tay DK. A clinical investigation of the efficacy of low level laser therapy in reducing orthodontic postadjustment pain. Am J Orthod Dentofac Orthop. 1995;108:614–22. 10.1016/s0889-5406(95)70007-2 7503039

[14] Carvalho FB, Barbosa AF, Zanin FA, Brugnera Júnior A, Silveira Júnior L, Pinheiro AL. Use of laser fluorescence in dental caries diagnosis: a fluorescence x biomolecular vibrational spectroscopic comparative study. Braz Dent J. 2013;24:59–63. 23657415 10.1590/0103-6440201302123

[15] Vitale MC, Sfondrini MF, Croci GA, Paulli M, Carbone L, Gandini P, et al. Diode laser-assisted surgical therapy for early treatment of oral mucocele in a newborn patient: case report and procedures checklist. Case Rep Dent. 2018;2018:3048429. 29854481 10.1155/2018/3048429 PMC5941754

[16] Galafassi D, Scatena C, Galo R, Curylofo-Zotti FA, Corona SAM, Borsatto MC. Clinical evaluation of composite restorations in er:yag laser-prepared cavities re-wetting with chlorhexidine. Clin Oral Investig. 2017;21:1231–41. 27376544 10.1007/s00784-016-1897-x

[17] Sfondrini MF, Calderoni G, Vitale MC, Gandini P, Scribante A. Is laser conditioning a valid alternative to conventional etching for aesthetic brackets? Eur J Paediatr Dent. 2018;19:61–6. 29569456 10.23804/ejpd.2018.19.01.11

[18] Merigo E, Rocca JP, Pinheiro ALB, Fornaini C. Photobiomodulation therapy in oral medicine: a guide for the practitioner with focus on new possible protocols. Photobiomodul Photomed Laser Surg. 2019;37:669–80. 31589560 10.1089/photob.2019.4624

[19] Harazaki M, Takahashi H, Ito A, Isshiki Y. Soft laser irradiation induced pain reduction in orthodontic treatment. Bull Tokyo Dent Coll. 1998;39:95–101. 9667142

[20] Baxter GD, Liu L, Petrich S, Gisselman AS, Chapple C, Anders JJ, et al. Low level laser therapy (Photobiomodulation therapy) for breast cancer-related lymphedema: a systematic review. BMC Cancer. 2017;17:833. 29216916 10.1186/s12885-017-3852-x PMC5719569

[21] de Pauli Paglioni M, Alves CGB, Fontes EK, Lopes MA, Ribeiro ACP, Brandao TB, et al. Is photobiomodulation therapy effective in reducing pain caused by toxicities related to head and neck cancer treatment? A systematic review. Support Care Cancer. 2019;27:4043–54. 31264186 10.1007/s00520-019-04939-2

[22] Glass GE. Photobiomodulation: the clinical applications of low-level light therapy. Aesthet Surg J. 2021;41:723–38. 33471046 10.1093/asj/sjab025

[23] Kang Y, Rabie AB, Wong RW. A review of laser applications in orthodontics. Int J Orthod Milwaukee. 2014;25:47–56. 24812743

[24] Walsh LJ. The current status of laser applications in dentistry. Aust Dent J. 2003;48:146–55. quiz 98. 14640367 10.1111/j.1834-7819.2003.tb00025.x

[25] Mirhashemi A, Rasouli S, Shahi S, Chiniforush N. Efficacy of photobiomodulation therapy for orthodontic pain control following the placement of elastomeric separators: a randomized clinical trial. J Lasers Med Sci. 2021;12:e8. 34084734 10.34172/jlms.2021.08 PMC8164901

[26] Celebi F, Turk T, Bicakci AA. Effects of low-level laser therapy and mechanical vibration on orthodontic pain caused by initial archwire. Am J Orthod Dentofac Orthop. 2019;156:87–93. 10.1016/j.ajodo.2018.08.021 31256846

[27] Eslamian L, Borzabadi-Farahani A, Hassanzadeh-Azhiri A, Badiee MR, Fekrazad R. The effect of 810-nm low-level laser therapy on pain caused by orthodontic elastomeric separators. Lasers Med Sci. 2014;29:559–64. 23334785 10.1007/s10103-012-1258-1

[28] Celebi F, Bicakci AA, Kelesoglu U. Effectiveness of low-level laser therapy and chewing gum in reducing orthodontic pain: a randomized controlled trial. Korean J Orthod. 2021;51:313–20. 34556585 10.4041/kjod.2021.51.5.313 PMC8461383

[29] Bart J, Fligner MA, Notz WI. Sampling and statistical methods for behavioral ecologists. Cambridge University Press; 1998.

[30] Bavbek NC, Tuncer BB, Tortop T, Celik B. Efficacy of different methods to reduce pain during debonding of orthodontic brackets. Angle Orthod. 2016;86:917–24. 27172508 10.2319/020116-88R.1 PMC8597343

[31] Sullivan MJ, Bishop SR, Pivik J. The pain catastrophizing scale: development and validation. Psychol Assess. 1995;7:524–32.

[32] Abtahi SM, Mousavi SA, Shafaee H, Tanbakuchi B. Effect of low-level laser therapy on dental pain induced by separator force in orthodontic treatment. Dent Res J (Isfahan). 2013;10:647–51. 24348624 PMC3858741

[33] da Cunha LA, Firoozmand LM, da Silva AP, Camargo SE, Oliveira W. Efficacy of low-level laser therapy in the treatment of temporomandibular disorder. Int Dent J. 2008;58:213–7. 18783114 10.1111/j.1875-595x.2008.tb00351.x

[34] Prasad SMV, Prasanna TR, Kumaran V, Venkatachalam N, Ramees M, Abraham EA. Low-level laser therapy: a noninvasive method of relieving postactivation orthodontic pain-a randomized controlled clinical trial. J Pharm Bioallied Sci. 2019;11:S228–31. 31198342 10.4103/JPBS.JPBS_303_18 PMC6555383

[35] Eslamipour F, Motamedian SR, Bagheri F. Ibuprofen and low-level laser therapy for pain control during fixed orthodontic therapy: a systematic review of randomized controlled trials and meta-analysis. J Contemp Dent Pract. 2017;18:527–33. 28621287 10.5005/jp-journals-10024-2078

[36] Wu S, Chen Y, Zhang J, Chen W, Shao S, Shen H, et al. Effect of low-level laser therapy on tooth-related pain and somatosensory function evoked by orthodontic treatment. Int J Oral Sci. 2018;10:22. 29967411 10.1038/s41368-018-0023-0 PMC6028457

[37] Lo Giudice A, Nucera R, Perillo L, Paiusco A, Caccianiga G. Is low-level laser therapy an effective method to alleviate pain induced by active orthodontic alignment archwire? A randomized clinical trial. J Evid Based Dent Pract. 2019;19:71–8. 30926104 10.1016/j.jebdp.2018.11.001

[38] Turhani D, Scheriau M, Kapral D, Benesch T, Jonke E, Bantleon HP. Pain relief by single low-level laser irradiation in orthodontic patients undergoing fixed appliance therapy. Am J Orthod Dentofac Orthop. 2006;130:371–7. 10.1016/j.ajodo.2005.04.036 16979496

[39] Fekrazad R, Golshah A, Shirazi ER. Efficacy of photobiomodulation therapy versus soft acrylic wafer for reduction of pain associated with orthodontic metal bracket removal: a clinical trial. Photobiomodul Photomed Laser Surg. 2022;40:463–71. 35766588 10.1089/photob.2021.0183

[40] Brook PH, Shaw WC. The development of an index of orthodontic treatment priority. Eur J Orthod. 1989;11:309–20. 2792220 10.1093/oxfordjournals.ejo.a035999

[41] Youssef M, Ashkar S, Hamade E, Gutknecht N, Lampert F, Mir M. The effect of low-level laser therapy during orthodontic movement: a preliminary study. Lasers Med Sci. 2008;23:27–33. 17361391 10.1007/s10103-007-0449-7

[42] Artés-Ribas M, Arnabat-Dominguez J, Puigdollers A. Analgesic effect of a low-level laser therapy (830 nm) in early orthodontic treatment. Lasers Med Sci. 2013;28:335–41. 22814893 10.1007/s10103-012-1135-y

[43] Duarte de Oliveira FJ, Brasil G, Araújo Soares GP, Fernandes Paiva DF, de Assis de Souza Júnior F. Use of low-level laser therapy to reduce postoperative pain, edema, and trismus following third molar surgery: a systematic review and meta-analysis. J Craniomaxillofac Surg. 2021;49:1088–96. 34217567 10.1016/j.jcms.2021.06.006

[44] Kiraly M, Bender T, Hodosi K. Comparative study of shockwave therapy and low-level laser therapy effects in patients with myofascial pain syndrome of the trapezius. Rheumatol Int. 2018;38:2045–52. 30171341 10.1007/s00296-018-4134-x

[45] Weber C, Thai V, Neuheuser K, Groover K, Christ O. Efficacy of physical therapy for the treatment of lateral epicondylitis: a meta-analysis. BMC Musculoskelet Disord. 2015;16:223. 26303397 10.1186/s12891-015-0665-4 PMC4549077

[46] Oliver RG, Knapman YM. Attitudes to orthodontic treatment. Br J Orthod. 1985;12:179–88. 3863673 10.1179/bjo.12.4.179

[47] Dundar U, Evcik D, Samli F, Pusak H, Kavuncu V. The effect of gallium arsenide aluminum laser therapy in the management of cervical myofascial pain syndrome: a double blind, placebo-controlled study. Clin Rheumatol. 2007;26:930–4. 17021664 10.1007/s10067-006-0438-4

[48] Guerreiro MYR, Monteiro LPB, de Castro RF, Magno MB, Maia LC, da Silva Brandão JM. Effect of low-level laser therapy on postoperative endodontic pain: an updated systematic review. Complement Ther Med. 2021;57:102638. 33307205 10.1016/j.ctim.2020.102638

[49] Camacho AD, Reyes MB, Cujar SAV. A systematic review of the effective laser wavelength range in delivering photobiomodulation for pain relief in active orthodontic treatment. Rev Orthop Dento Fac. 2020;18(4):684–95. 10.1016/j.ortho.2020.08.008 33060065

[50] Caccianiga P, Carminati I, Caccianiga G. Photobiomodulation with laser technology to reduce pain perception during fixed orthodontic treatment: literature review and new perspectives. Inventions. 2023;8(1):46.

[51] Rumão WL, Valdrighi HC, Furletti VF. Influence of photobiomodulation on pain perception during initial orthodontic tooth movement. Rev Odontol UNESP. 2020;49:e20200003.

[52] Hmida H, Qalalwa M, Amor WB, Dallel I. Efficacy of photobiomodulation in orthodontic pain management: a systematic review of literature. Saudi J Oral Dent Res. 2025;10(1):67–78.

[53] Wang X, Liu Q, Peng J, Song W, Zhao J, Chen L. The effects and mechanisms of PBM therapy in accelerating orthodontic tooth movement. Biomolecules. 2023;13(7):1140. 37509176 10.3390/biom13071140 PMC10377711

